# The Relationship between Bicultural Identity Integration, Self-Esteem, Academic Resilience, Interaction Anxiousness, and School Belonging among University Students with Vocational Qualifications

**DOI:** 10.3390/ijerph19063632

**Published:** 2022-03-18

**Authors:** Wenxin Chen, Yi Lin, Xiaoyan Yu, Wen Zheng, Shiyong Wu, Mingxi Huang, Wei Chen, Shuyi Zhou

**Affiliations:** 1 School of Education, South China Normal University, Guangzhou 510631, China; 2021020688@m.scnu.edu.cn (W.C.); xiaoyan.yu@m.scnu.edu.cn (X.Y.); huangmingxi@m.scnu.edu.cn (M.H.); 2 Social Science Office, South China Normal University, Guangzhou 510631, China; linyi@m.scnu.edu.cn; 3 School of Education, Huizhou University, Huizhou 516000, China; zhengwen@hzu.edu.cn; 4 South China Vocational Education Research Centre, South China Normal University, Foshan 528225, China; 5 Faculty of Foreign Language, Dongguan Science & Technology School, Dongguan 523470, China; 2019023366@m.scnu.edu.cn

**Keywords:** bicultural identity integration, self-esteem, academic resilience, integration anxiousness, school belonging, transition, vocational pathway university students

## Abstract

Background: University students with a vocational pathway face greater cultural, psychological, cognitive, and behavioral challenges during the transition process than their counterparts with an academic route. Method: This study examined the predictive effect of bicultural identity integration, self-esteem, academic resilience, and interaction anxiousness on school belonging using a quantitative approach with 326 Chinese vocational pathway university student participants. Result: The participants had high levels of cultural adaptability, self-esteem, academic resilience, and school belonging, but they also displayed moderate interaction anxiousness. Bicultural identity integration (*B* = 0.24; *p* < 0.001), self-esteem (*B* = 0.35; *p* < 0.001), and academic resilience (*B* = 0.25; *p* < 0.001) significantly positively predicted school belonging, while interaction anxiousness (*B* = −0.17; *p* < 0.01) negatively predicted school belonging. Conclusions: Bicultural identity integration, self-esteem, academic resilience, and interaction anxiousness were crucial determinants of school belonging among Chinese university students with vocational qualifications. Effective measures should be initiated to boost their feelings of being recognized, respected, and connected to the university community.

## 1. Introduction

The transition trajectories and experiences of university students with vocational qualifications have received considerable attention from policymakers, educators, and scholars over the past decade [1,2,3,4]. As part of the educational diversity, vocational education and training students participating in higher education are viewed as enablers to increase social class mobility and reduce dynamic social inequality [5,6]. Accessing higher education via vocational pathways can help students enhance their employability-related skills and realize their career expectations. However, due to the significant differences in curriculum, teaching, and examination between the higher education system and the vocational education system [7], students progressing to university from vocational education are more likely to experience transitional frictions in episteme and pedagogy [8] compared to their peers who upgrade to higher education from a standard academic pathway. In addition to feeling their learning abilities are insufficient, these students are also more prone to feel confused about integrating their bicultural identity [9] and suffer from emotional tension and anxiety [8,10,11]. Empirical studies have found that more than 20% of university students with vocational education backgrounds feel highly anxious and depressed in adapting to new learning patterns [12]. These passive transition experiences could lead to a lack of self-worth [13] and a sense of separation from the university community, further impacting their transition success. Conversely, students who have developed different types of social networks with new friends in the new environment and who have become embedded into their university life are more likely to develop greater levels of self-esteem and optimism and then gain a sense of belonging [2]. Additionally, students’ demographical characteristics, such as gender, age [14], socioeconomic status [15], and their means of entering university [1], have been associated with their transition achievement.

The relationships between the transition challenges in the cultural, psychological, learning, and interacting aspects and transition outcomes in connecting to the community are also evident from the research on school belonging. School belonging was defined by Goodenow and Grady [16] as “the extent to which students feel personally accepted, respected, included, and supported by others in the school social environment”. Among the factors influencing students’ sense of school belonging, cultural, academic, and psychosocial determinants have been well documented [17]. School belonging reflects students’ positive attachment to a group to improve their interpersonal integration and academic level [18]. Individuals with a high sense of school belonging are more likely to report psychological benefits, such as increased self-esteem [19], reduced interaction anxiety or depression [20], and positive life transition [21]. Self-esteem means that students can understand and manage their emotions with great detail and insight [22], while interaction anxiousness refers to a negative emotion caused by dissatisfaction with one’s existing social state and difficulty integrating into the group [2].

Moreover, previous studies have identified that school belonging is closely related to bicultural identity integration and academic resilience [3,23]. Bicultural identity integration usually occurs when people maintain the values and customs of their native culture and recognize those of mainstream society [24,25,26,27]. In the school setting, bicultural identity integration indicates the process and ability to familiarize oneself with diverse academic organizational cultures and tracks. Students with a high bicultural identity integration ability can more easily form positive intermembership [26] and achieve a stronger sense of school belonging [27,28]. Academic resilience refers to students’ capacity to achieve good learning outcomes despite a nonprivileged background [29]. Students without the appropriate sense of belonging can have difficulties achieving academic success [30].

Based on these previous studies, it is reasonable to infer that the factors influencing the transition process and success of university students with vocational pathways can be categorized into three interconnected dimensions—personal background, culture–psychology, and learning–interacting—and that school belonging theory is applicable to transition research. Namely, school belonging can be used as an aggregate variable to assess whether students have successfully embedded into their university life. Relatively, the four key factors predicting school belonging—bicultural identity integration, self-esteem, academic resilience, and interaction anxiousness—can be linked to the culture–psychology and learning–interacting dimensions. Subsequently, they can be employed to evaluate the challenges faced by students in their transition processes.

However, most existing studies on the experiences and performances of vocational pathway university students have utilized a phenomenological qualitative approach, and the cultural, psychological, cognitive, and behavioral determinants affecting transition success have not yet been synthetically identified from the perspective of school belonging. Specifically, little attention has been paid to the interrelations between bicultural identity integration, self-esteem, academic resilience, and interaction anxiousness, alongside their predictive roles in school belonging. More importantly, despite considerable concerns about students’ transition experiences from vocational colleges to universities in the United States, Canada, the U.K., and Australia [4,14,31,32,33], they remain relatively underexplored in the Chinese university setting. Therefore, there is great significance in holistically investigating the influence of bicultural identity integration, self-esteem, academic resilience, and interaction anxiousness on school belonging among vocational pathway university students.

**Figure 1:**
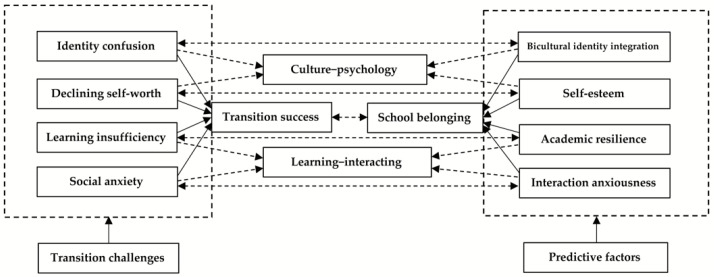
Conceptual model. The solid right icon arrow means the influence of summarized transition challenges on transition success. The solid left icon arrow implies the impact of extracted key factors on school belonging. The single dashed arrow points to the categorized dimension. The double dashed arrow indicates the inter-relationship between observed variables and transition challenges.

With this in mind, we designed an integrated conceptual model to examine the transition process and outcomes of Chinese university students with a vocational route, in which the four crucial factors—bicultural identity integration, self-esteem, academic resilience, and interaction anxiousness—were extracted as independent variables to explore their predictive effects on the dependent variable of school belonging (Figure 1). In addition, the personal background factors were considered as group variables to validate the model. This study will add value to the theoretical literature on transition theory and empirical research on university students’ wellbeing and mental health by assessing the complex interplay between school belonging and its correlations in a sample composed of Chinese students with vocational qualifications. The following hypotheses were posited:

**Hypothesis** **1** **(H1).** *Bicultural identity integration positively predicts school belonging.*

**Hypothesis** **2** **(H2).** *Academic resilience positively predicts school belonging.*

**Hypothesis** **3** **(H3).** *Self-esteem positively predicts school belonging.*

**Hypothesis** **4** **(H4).** *Interaction anxiousness negatively predicts school belonging.*

## 2. Materials and Methods

### 2.1. Participants

The participants consisted of 326 higher education students who had obtained a vocational college degree and were currently studying at a research-intensive university. More female than male students were included in this study (64% vs. 36%). Participants’ ages ranged from 17 to 29 years (*M* = 20.39, *SD* = 1.79). Approximately 59% of the participants came from rural areas. The majority of the participants (approximately 93%) upgraded directly from higher vocational college, while the remaining experienced a two-wave transition from a secondary vocational school to a higher vocational college and, ultimately, to university. The disciplines that the participants enrolled in were Business English (29.8%), Electronic Commerce (36.5%), and Internet Engineering (33.7%). They were all in their first year of study at South China Normal University, which recruits upgraded students from higher vocational colleges to university. More detailed demographic information can be found in Table 1.

### 2.2. Measures

#### 2.2.1. Bicultural Identity Integration Scale

Bicultural identity integration was assessed with the Chinese version of the Bicultural Identity Integration Scale–Version 1 (BIIS–1), originally constructed by Benet-Martinez and Haritatos [27], consisting of eight items that examine “how people deal with bicultural identity” [27,34,35]. The BIIS-1 involves identity conflict (four items) and identity distance (four items). Responses are rated on a seven-point Likert-type scale (1 = strongly disagree to 7 = strongly agree). The participants were asked to rate their level of integration with statements such as “I am trapped between two cultures”. Higher total scores indicate higher bicultural identity integration. The internal consistency coefficients of the Chinese version ranged from 0.62 to 0.83 (see [36,37,38]). In this study, Cronbach’s alpha coefficient was 0.64.

#### 2.2.2. Self-Esteem Scale

Self-esteem was gauged with the Chinese version of the Self-Esteem Scale (SES), originally constructed by Rosenberg [39] and translated by Han et al. [40], including ten items. The participants were required to rate their agreements with statements such as “I think I am a valuable person” on a seven-point Likert-type scale (1 = strongly disagree to 7 = strongly agree) [41]. Higher scores demonstrate a stronger sense of self-esteem. Cronbach’s alpha for the Chinese version ranged from 0.77 to 0.87 [42,43,44,45]. In this study, Cronbach’s alpha was 0.90.

#### 2.2.3. Academic Resilience Scale

Academic resilience was calculated with a six-item Chinese version of the Academic Resilience Scale (ABS), initially developed by Martin and Marsh [46]. The responses were marked on a seven-point Likert-type scale (1 = strongly disagree to 7 = strongly agree). The participants were asked to rank their level of agreement with statements such as “I think I am good at dealing with schoolwork pressures”. Higher scores indicate better academic resilience. Cronbach’s alpha coefficient of the Chinese version was 0.87 [47]. In this study, Cronbach’s alpha was 0.92.

#### 2.2.4. Interaction Anxiousness Scale

Interaction anxiousness was measured with the Chinese version of the Interaction Anxiousness Scale (IAS), initially proposed by Leary [48] and reformulated by Peng et al. [49], comprising 15 items. The respondents were asked to rate their degree of anxiety in social interactional situations on a seven-point Likert-type scale (1 = absolutely incorrect to 7 = absolutely correct). A sample item is “I wish I had more confidence in social occasions.” Higher scores signify higher interaction anxiousness. Cronbach’s alphas of the Chinese version ranged from 0.81 to 0.86 [50,51,52]. In this study, Cronbach’s alpha reliability coefficient was 0.87.

#### 2.2.5. School Belonging Scale

School belonging was evaluated with the Chinese version of the School Belonging Scale (SBS), developed by Zhu and Han [53], comprising five items that were extracted from the scales of Bollen and Hoyle [54] and Anderman [55]. The responses were rated on a seven-point Likert-type scale (1 = strongly disagree to 7 = strongly agree). The participants were asked to rate their agreements with statements such as “I am interested in what happened at school”. Higher total scores mean stronger feelings of school belonging [17]. Cronbach’s alpha coefficient of the Chinese version was 0.87 [53]. In the present study, Cronbach’s alpha coefficient was 0.91.

### 2.3. Procedure

Data were gathered via the online survey software Wenjuanxing, known as Chinese Qualtrics, so that the participants could respond to the survey anywhere and anytime. The survey was distributed in a course taught by the corresponding author responsible for training the participants’ knowledge and skills of designing and filling in a questionnaire to enhance the response quality because of the particularity and rarity of the samples. Data were collected between 5 and 12 January 2021, which was when the students had completed a half-year of university learning and experiences. After all participants had completed the questionnaire, the data were strictly screened regarding whether they contained random or incongruent responses, such as an extremely short response time and/or repetitive answers, which were removed. Eventually, 326 highly valid questionnaires were retained for further analyses.

### 2.4. Data Analysis

The data were analyzed with an online data analysis platform, SPSSAU, accessed at http://spssau.com/index.html on 25 December 2021, developed with the same kernel algorithm as SPSS statistical software designed by the American International Business Machines Corporation (IBM) headquartered in Armonk, New York. First, common method variance was gauged to evaluate the data validity using Harman’s single-factor test [56]. The first factor extracted accounted for 22.31% of the total variance, less than 40% of the recommended value [57], indicating that the common method bias was not considered a prominent issue in this study. The absolute values of kurtosis (close to 2, less than the suggested 10 or 7) and skewness (approximately 1, less than the advised 5 or 2) also indicated that the data observed in this study were normally distributed [58,59]. Secondly, descriptive statistics were employed to analyze the variables’ mean, standard deviation, and differences. Next, bivariate correlations were conducted to judge the associations among variables. Finally, regression analysis was performed to evaluate the posited research hypotheses. The demographic characteristics of the participants, such as gender, residency, upgrade, and discipline, were considered dummy variables for grouped regression analysis to assess the robustness of the model, and the base variables were coded as 0, as indicated in Table 1. For the discipline, Business English and Electronic Commerce were collapsed into Social Science and assigned as the base variable.

### 2.5. Ethics

This study was examined and authorized by the South China Normal University Academic Ethics Committee. The research ethics approval was attached to the explanatory statement, and a consent form was supplied to the participants.

## 3. Results

### 3.1. Descriptive Statistics

Table 2 presents the means, standard deviations, and significant differences between gender, residency, upgrade, and discipline. Overall, the participants’ means in all variables were higher than the median (*M* > 4), especially for school belonging (*M* > 6), which was close to the maximum. Regarding gender differences, except for academic resilience, the female participants scored higher for bicultural identity integration, interaction anxiousness, self-esteem, and school belonging than the males, but the differences were only significant for self-esteem and academic resilience (*p* < 0.05). In terms of residency, the participants from the city had higher scores for all observed variables, but no significant difference was found regarding family background. Regarding upgrades, participants who had a vocational college to university route gained higher scores for academic resilience, bicultural identity integration, self-esteem, and school belonging, but there were only significant differences for the latter three variables (*p* < 0.05, 0.01, and 0.001, respectively). In terms of discipline, participants majoring in Business English gained the highest scores for interaction anxiousness, academic resilience, and school belonging, while those studying Electronic Commerce had the highest scores for bicultural identity integration and self-esteem; namely, participants from Social Science disciplines obtained higher scores in all variables than their counterparts from Internet Engineering, but the differences were only significant for self-esteem and school belonging (*p* < 0.01).

### 3.2. Correlation Analysis

Table 3 shows the correlations of the variables investigated. All the constructs were significantly correlated regarding the bivariate correlations among the observed constructs. The correlations were significantly positive for bicultural identity integration, self-esteem, academic resilience, and school belonging (*p* < 0.01). Interaction anxiousness was significantly negatively related to the other constructs (*p* < 0.01). Notably, self-esteem was strongly correlated with academic resilience and school belonging (r > 0.5, *p* < 0.01) and moderately correlated with bicultural identity integration and interaction anxiousness (0.3 < r < 0.5, *p* < 0.01) [60]. Regarding the correlations between control variables and constructs, gender was weakly related to self-esteem and academic resilience (r < 0.3, *p* < 0.05); both upgrade and discipline were weakly related to bicultural identity integration, self-esteem, and school belonging (r < 0.3, *p* < 0.01). Interestingly, residency was not significantly related to the other variables.

### 3.3. Regression Analysis

Table 4 displays the regression results for school belonging. Gender was not a significant predictor, nor were residency or discipline, other than upgrade. A strong association was reported for bicultural identity integration, with higher levels predicting a stronger feeling of school belonging, supporting Hypothesis 1. Similarly, self-esteem and academic resilience were positively associated with a strong sense of school belonging, supporting Hypotheses 3 and 4. Furthermore, interaction anxiousness was negatively predictive of school belonging, supporting Hypothesis 2. Therefore, all the hypotheses were fully supported.

To further test the equality between two linear regressions across groups, the Chow Test [61] was performed to determine whether changes occurred between two samples of control variables. The results in Table 5 indicate a significant mutation effect between two subgroups of the upgrade compared to the other control variables. Students who upgraded via the higher vocational college to university route had a stronger feeling of school belonging than their peers progressing via a secondary vocation school to higher vocational college to university pathway. This result further confirms that the upgrade significantly impacts school belonging rather than other individual characteristics.

## 4. Discussion

In this study, we examined the predictive effect of bicultural identity integration, interaction anxiousness, self-esteem, and academic resilience on school belonging among university students with vocational qualifications in the process of transitioning to higher education. The participants’ overall means were above the midpoint of the scales, indicating that they were highly adapted to the new school culture and teaching and learning approaches, with a strong sense of identity recognition, academic engagement, and self-respect. Remarkably, they had prominent scores in school belonging, indicating that they were highly proud of being a member of the university they had enrolled in. This result could be explained by the fact that they were very satisfied with and proud of their successful progression to a top-tier university as a misrecognized vocational student at the bottom of the education hierarchy [62]. However, the participants were moderately anxious to contact their academic peers who directly entered higher education from high school. These results are in line with the findings that, despite perceiving themselves as being agentic and performative in completing learning tasks and other activities, they internally see themselves as inferior or inadequate due to the stereotyped attitude of “stupid and lazy” in society [13,63]. Therefore, they are unconfident, or even avoidant, when connecting with their academic counterparts. These results are also consistent with the finding indicating that students transitioning to tertiary education from the vocational education system usually feel overwhelmed and anxious about their success at university [3].

The significant difference analysis revealed that female students’ self-esteem is stronger than male students. This result contradicts the common assertion that male students have higher self-esteem than female students [64,65]. This result is also unlike the finding that male students have higher self-esteem after transitioning to university from secondary school than female students [66]. However, female students with highly positive self-esteem were less resilient than male students when confronting academic challenges. This result is contrary to the empirical finding that female transfer students perform better academically than males at university [14]. This result is also incongruent with the finding that students with high self-esteem tend to have lower academic stress, higher academic self-efficacy, better academic achievements, and stronger academic resilience [67]. This result may be explained by the fact that academic resilience is only one way of gaining self-esteem, and other determinants, such as cultural and demographic factors, are also influential [68].

Moreover, students who entered university via the higher vocational college pathway had significantly higher bicultural identity integration, self-esteem, and school belonging levels. This result aligns with the finding that joint students, referring to those who attend a higher vocational college and application-oriented university, have strong feelings of cultural recognition and school belonging [69]. This result may be supported by the fact that many students on a higher vocational college to university route obtained a relatively high score in the Nationwide Unified Examination for Admissions to General Universities and Colleges. Unfortunately, they were not admitted to the general university that they applied for and were unwilling to enroll in a university ranking below their preferred one. Alternatively, they chose to continue studying at a top-ranking higher vocational college. Therefore, they have a strong sense of self-worth and self-esteem regarding their academic performance and are well-adapted to the new culture and community.

Furthermore, students who majored in the Social Science discipline had significantly higher self-esteem and school belonging levels. This result supports the finding that students’ sense of self-esteem and school belonging is associated with their willingness to consider majoring in a discipline [70], yet it is contrary to the conclusion that there are no significant differences in the fields of self-esteem and school belonging among university students [71]. This result may be due to the type of university they are currently attending, which is well known for Social Science disciplines. Hence, students choosing Business English and Electronic Commerce majors built a stronger sense of self-esteem regarding their enrollment and belonging to the university.

In addition, the results presented close links between bicultural identity integration, interaction anxiousness, self-esteem, and academic resilience among Chinese university students with college degrees. These results align with the findings that students who can better integrate two identities have more time and energy to devote to learning, higher creativity, better team performance, and less sensitivity to bias [26] and that their academic resilience is improves [26,72,73]. These results are also similar to the findings that students with a more positive attitude towards themselves have higher academic resilience [74]. Significantly, interaction anxiousness was negatively associated with bicultural identity integration, self-esteem, and academic resilience. The higher students’ interaction anxiousness was, the more confused they were about their identities, the lower their self-esteem, and the less outstanding their academic performance was. This result provides evidence that higher education students switching from a vocational system with high interaction anxiousness tended to resist the transformation process, affecting their engagement and performance in many facets of the university journey [12,32].

The regression results also identified the significant impacts of bicultural identity integration, interaction anxiousness, self-esteem, and academic resilience on school belonging among Chinese university students with vocational qualifications. The following subsections detail the specific predictive effect of the four observed variables on school belonging.

### 4.1. The Relationship between Bicultural Identity Integration and School Belonging

The results showed that bicultural identity integration has a significantly positive effect on school belonging, fully supporting Hypothesis 1. This indicates that the higher the bicultural identity integration levels of university students with vocational qualifications were, the higher their sense of school belonging was.

This result is consistent with the previous theoretical argument [1,18] that university students from a vocational school face severe challenges in integrating their dual identities. According to the transition theory proposed by Schlossberg [75], students entering a new learning environment are likely to struggle with transition shock, including culture shock [1,25], defined by the identity misrecognition that results from losing familiar signs and symbols of social intercourse [76]. The most appropriate concept of transition is the process of identity development and alteration, which changes over time through complex interactions between students, faculty, staff, and the institutional environment [77]. This transition is not merely a geographical migration, away from friends and family, but a cultural integration [78]. When students who maintain a strong sense of recognition of their original culture establish connections with members of other groups, they may be confronted with cultural conflict and cultural distance [24,25]. Cultural conflict affects the efficacy of bicultural students in maintaining a consistent and harmonious self-image and sense of group belonging [72]. Cultural distance affects the effectiveness of biculturalists in creating a collective and collaborative cultural identity. In general, harmonious identity integration allows transitioners to perceive fewer stressors, have lower neuroticism, and achieve a stronger sense of belonging [24]. Therefore, students with high bicultural identity integration are more likely to form positive intergroup relationships and a strong sense of school belonging [26]. This result is in line with Lazarowicz’s findings that most transfer students feel that being exposed to a different institutional culture is beneficial for adapting to new settings and forming new identities [31]. Based on these findings, it can be inferred that bicultural identity integration is a vital predictor of school belonging and should be considered when seeking to improve levels of school belonging among university students with vocational qualifications.

This result highlights empirical implications for enhancing students’ sense of school belonging by promoting their capacity to integrate their bicultural identity. Individually, students should be more proactive in building contacts with their new surroundings to develop their bicultural identity integration. Institutionally, the university should provide more guidance and opportunities to help students transition to the new environment, including transitional experience sharing, peer tutoring services [3], and cultural adaption courses. Through these activities, students can develop cultural self-confidence, improve their communication skills, and increase their enthusiasm and sense of belonging to the school.

### 4.2. The Relationship between Self-Esteem and School Belonging

The results also demonstrated that self-esteem significantly positively predicts school belonging, fully supporting Hypothesis 3. The higher the level of students’ self-esteem was, the stronger their sense of school belonging was.

This result confirms the theoretical assumptions that students’ self-worth contributes to the feeling of school belonging [79], and a positive self-evaluation is viewed as promoting a positive school connection [80]. This result further provides evidence for the hypothesis of Taylor et al. [81] that an individual’s attitude toward the self, such as self-esteem, promotes their academic success and wellbeing, which are critical components of school belonging. This result is similar to the empirical finding that students who view themselves as successful transitioners are more inclined to feel involved in the classroom and university community [31]. This result also resonates with the empirical finding of Perry and Lavins-Merillat [82] that growth in self-esteem among young people leads to an enhancement in school belonging. Their affirmation of themselves will be projected into the school, and their sense of belonging will also increase.

In contrast, when transitioning across the different schools, students’ declining self-esteem may decrease their sense of school belonging [83]. Existing research has outlined that vocational students stepping into university are more likely to position themselves negatively during the progression than their academic peers [63]. Due to the transitional friction associated with cognitive doubts and teaching tension, along with emotional tension and anxiety, students are prone to disvalue their professional knowledge and learning achievement. These frustrated transitional experiences lead to a low level of school belonging. Therefore, universities should make students feel respected, valued, cared for, and liked by others to enhance their healthy self-esteem in order to deal more flexibly with various sharp transitional challenges and improve their levels of school belonging.

### 4.3. The Relationship between Academic Resilience and School Belonging

The results indicated that academic resilience is significantly predictive of school belonging, fully supporting Hypothesis 2. This suggests that the higher students’ academic resilience was, the stronger their sense of belonging to the university became.

This result further provides empirical evidence for the school belonging argument that academic success is a salient predictor of students’ connectedness to their school [17]. Learning shock, defined as the experiences of confusion and anxiety that students suffer from exposure to unfamiliar learning and teaching approaches [22], was another essential element influencing students’ mental affiliation in transitioning to a university. According to the transition theory, students moving to an unfamiliar setting typically encounter cognitive and behavioral challenges, especially in the university context [1]. Transfer students who see the new learning context as a positive stimulus responded to academic challenges with resilience [32]. Considering this together with cultural factors, students with high bicultural identity integration have better academic resilience, defined by stronger cognitive processing ability and less cognition complexity, and, subsequently, stronger school belonging [26]. Students encountering difficulties in adapting to the university pedagogy and learning methods are less likely to achieve academic success and feel that they belong to the university community [30]. This result is also supported by Najam’s [84] finding that college students’ academic resilience had a positive predictive effect on their school belonging.

Moreover, studies have shown that the school environment is a salient determinant in predicting students’ school belonging [85]. Learning is an interdependent process and cannot take place away from the study context [86]. Transfer students do not necessarily perceive themselves as lacking knowledge; rather, they do not gain the knowledge expected in the university [33]. In this context, universities and teachers should offer more academic support to facilitate students’ academic performance and foster their sense of school belonging by creating an effective learning atmosphere, promoting mastery goal orientation in the classroom, and applying proper academic pressure on their professional learning [87]. Individually, students should ease into the academic transition by taking a light-to-heavy strategy, such as a lower extracurriculum load in the first semester, to build and promote their academic resilience [31].

### 4.4. The Relationship between Interaction Anxiousness and School Belonging

The results revealed that interaction anxiousness significantly negatively predicts school belonging among Chinese university students with vocational qualifications, fully supporting Hypothesis 4. This result shows that the higher their interaction anxiousness level was during the transition period, the lower their sense of school belonging became.

This result acknowledges the importance of interpersonal communication and interaction in shaping and increasing the sense of psychological and affirmative belonging to a school in the transitional process. As Meehan and Howells pointed out, the relationship or sense of connection between students and others is essential for college life and crucial in the successful transition to higher education [18]. Students entering a new school familiarize themselves with learning methods and define their identities in the university community [2]. Once they fail to integrate the two distinct learning styles and cultural identities effectively, they may feel anxious and distressed when participating in social activities and may even avoid social connection. Those who report more connections with their peers have better academic achievement and a stronger sense of school belonging [28]. Conversely, university students with high social isolation and anxiety have poorer academic performance and reduced individual wellbeing [88]. Students with lower levels of social involvement with peers and friends may experience a weaker sense of school belonging [89]. For vocational students entering a new environment, maintaining close contact with at least one peer or social group brings meaningful benefits. Establishing friendships with different people can provide helpful advice and emotional support for first-year students. For example, building a social network with stakeholders can even provide valuable and applicable information about opportunities for school activities [2]. If the demand for this connection cannot be met, then interaction anxiousness will occur. Interaction anxiousness is also the internal motivation to develop school belonging. The emergence of interaction anxiousness reflects students’ greater eagerness to integrate into the new school life and a higher demand for school belonging. Anxious vocational pathway university students are more eager to be accepted, respected, and included by others and communities [18].

Therefore, some measures can be considered to help students better cope with the interaction anxiousness caused by integration and increase their feelings of school belonging. At the institutional level, universities should implement inclusive curriculum models, such as academic preparation seminars, welcome packs for vocational pathway students, and vocational pathway student peer contact programs so that learners who enter from different channels can successfully participate [90]. At the same time, the pedagogy should be significantly adjusted, focusing more on the training of practice-oriented knowledge and skills rather than purely rational training. At the individual level, students should proactively join clubs and societies to help establish social networks with their peers to reduce transition friction and enhance their sense of school belonging [91].

## 5. Limitations and Further Work

While the four hypotheses were verified in the present study, there are two limitations that must be acknowledged. The first limitation is that the data were gathered by self-reporting and are limited to only one top-ranking university. Therefore, causality and generalization in the results should be treated with caution yet may suggest common scenarios among vocational pathway university student cohorts. Further research is needed to cover a broader population from different universities that recruit vocational students to improve the generalizability and applicability of the study. Another limitation is that we mainly investigated the linear impact of the selected variables on school belonging. Future work should design a holistic model to examine the mechanisms of the interplay among variables, such as a moderation or mediation model. Moreover, other variables closely related to school belonging, such as cultural intelligence and imposter syndrome, should be included in future work, which would enrich the study of the transitional experiences and outcomes of university students with vocational qualifications.

## 6. Conclusions

Combining transition and school belonging theory, this study developed a comprehensive conceptual model to investigate the transition experiences and success among university students with a vocational pathway in their early transitional process. In this framework, the four essential factors related to cultural–psychological and learning–interacting dimensions—bicultural identity integration, self-esteem, academic resilience, and interaction anxiousness—were extracted to assess their predictive effect on school belonging. The results revealed that vocational-route university students have relatively high levels of bicultural identity integration, self-esteem, academic resilience, and school belonging. At the same time, they also feel highly anxious when interacting with others. The results also indicated that bicultural identity integration, self-esteem, and academic resilience significantly and positively predict school belonging, while interaction anxiousness has a significantly and negatively predictive effect on school belonging. Moreover, the upgrade pathway from higher vocational college to university also significantly positively impacts students’ sense of school belonging. These empirical results add value to the current literature regarding transition theory and school belonging research on how cultural–psychological and cognitive–behavioral factors influence students’ mental health in the context of transitioning from a vocational system to a university. This study also provides informative insights into how to accelerate vocational pathway university students’ bicultural identity integration, self-esteem, and academic resilience; reduce their interaction anxiousness; and, ultimately, boost their sense of school belonging by offering transition guidance, reforming curriculum content and teaching methods, holding interesting student events, and deepening collaboration with vocational schools. This study could be further strengthened and generalized by amplifying the sample diversity and clarifying the mechanism of the interplay among variables.

## Figures and Tables

**Table 1:** Characteristics of the study participants.

Characteristics	Subcharacteristics	Number	Percentage
Gender	Male (0)	117	35.9%
	Female	209	64.1%
Age	17–20 years	213	65.3%
	21–25 years	108	33.1%
	26–29 years	5	1.6%
Residency	Rural (0)	191	58.6%
	Urban	135	41.4%
Upgrade	College to university (0)	304	93.3%
	School to college to university	22	6.7%
Discipline	Business English (0)	97	29.8%
	Electronic Commerce (0)	119	36.5%
	Internet Engineering	110	33.7%

Number 0 in subcharacteristics means the base variable coded. Business English and Electronic Commerce were collapsed into Social Science and assigned as the base variable.

**Table 2:** Means, standard deviations, and significant differences among variables in gender, residency, upgrade, and disciplines.

Variables	M	SD	Male (*n* = 117)		Female (*n* = 209)		*Sig.*	Rural (*n* = 191)		Urban (*n* = 135)		*Sig.*	College-University (*n* = 304)		School-College-University (*n* = 22)		*Sig.*	Business English (*n* = 97)		Electronic Commerce (*n* = 119)		Internet Engineering (*n* = 110)		*F*
			M	SD	M	SD		M	SD	M	SD		M	SD	M	SD		M	SD	M	SD	M	SD	
Bicultural identity integration	4.52	0.65	4.45	0.69	4.56	0.62	0.16	4.47	0.65	4.59	0.64	0.12	4.54	0.63	4.21	0.79	0.02 *	4.52	0.56	4.59	0.69	4.44	0.67	0.26
Self-esteem	5.39	0.97	5.23	0.99	5.48	0.96	0.02 *	5.34	1.00	5.46	0.93	0.30	5.43	0.95	4.85	1.13	0.01 **	5.51	0.99	5.54	0.90	5.12	0.99	0.01 **
Academic resilience	5.08	1.12	5.25	1.12	4.98	1.11	0.03 *	5.01	1.13	5.17	1.10	0.22	5.09	1.12	4.88	1.14	0.39	5.17	1.06	5.10	1.13	4.97	1.15	0.42
Interaction anxiousness	4.19	0.85	4.07	0.90	4.26	0.82	0.05	4.19	0.77	4.19	0.96	0.99	4.19	0.84	4.31	1.05	0.51	4.22	0.82	4.21	0.85	4.16	0.89	0.85
School belonging	6.02	0.95	5.99	0.99	6.04	0.94	0.69	5.94	1.02	6.14	0.84	0.06	6.09	0.93	5.14	0.88	0.00 ***	6.18	0.89	6.10	0.86	5.80	1.07	0.01 **

* *p* < 0.05, ** *p* < 0.01, *** *p* < 0.001; *n*: number; M, mean; SD, standard deviation.

**Table 3:** Correlations of the variables.

Variables	1	2	3	4	5	6	7	8
1. Gender	−							
2. Residency	−0.09	−						
3. Upgrade	−0.18 **	0.07	−					
4. Discipline	−0.41 **	−0.01	0.26 **	−				
5. Bicultural identity integration	0.08	0.09	−0.13 *	−0.05	−			
6. Self-esteem	0.13 *	0.06	−0.15 **	−0.16 **	0.46 **	−		
7. Academic resilience	−0.12 *	0.07	−0.05	−0.07	0.29 **	0.56 **	−	
8. Interaction anxiousness	0.11	0.00	0.04	−0.03	−0.28 **	−0.38 **	−0.40 **	−
9. School belonging	0.02	0.11	−0.25 **	−0.16 **	0.39 **	0.56 **	0.16 **	−0.50 **

* *p* < 0.05, ** *p* < 0.01. The numbers 1, 2, 3, 4, 5, 6, 7, and 8 in the first line indicate the variables with the same numbers in the first column.

**Table 4:** Regression analysis for school belonging.

Variables	*B*	SE	β
Gender	−0.12	0.10	−0.06
Residency	−0.12	0.08	0.06
Upgrade	−0.65	0.17	−0.17 ***
Discipline	−0.08	0.10	−0.04
Bicultural identity integration	0.24	0.07	0.16 ***
Self-esteem	0.35	0.06	0.36 ***
Academic resilience	0.25	0.05	0.29 ***
Interaction anxiousness	−0.17	0.05	−0.15 **
R^2^	0.44		
Adjusted R^2^	0.42		

** *p* < 0.01, *** *p* < 0.001; SE, standard error.

**Table 5:** Chow Test for the influence of the grouped control variable on the regression model.

Control Variable	Sum of Squared Error			F	*p*
	Full Formula	Group 1	Group 2		
Gender	155.48	60.41	92.63	0.84	0.54
Residency		96.01	57.34	0.73	0.63
Upgrade		133.88	13.53	2.87	0.01 **
Discipline		83.78	70.27	0.49	0.82

** *p* < 0.01. Group 1 indicates the data grouped by male gender, rural area, college to university pathway, and Social Science discipline. Group 2 indicates the data grouped by female gender, urban area, school to college to university pathway, and Natural Science discipline.

## Data Availability

The data that support the findings of this study are available from the corresponding author with the permission of South China Normal University upon request. Restrictions apply to the availability of these data, which were used under license for this study.
